# Predictive Factors Associated With Methods of Suicide: The Korean National Investigations of Suicide Victims (The KNIGHTS Study)

**DOI:** 10.3389/fpsyt.2021.651327

**Published:** 2021-05-12

**Authors:** Hyewon Kim, Yuwon Kim, Gusang Lee, Jin Hwa Choi, Vidal Yook, Myung-Hee Shin, Hong Jin Jeon

**Affiliations:** ^1^Department of Psychiatry, Hanyang University Medical Center, Seoul, South Korea; ^2^Department of Data Science, Evidnet, Seongnam-si, South Korea; ^3^Korea Psychological Autopsy Center (KPAC), Seoul, South Korea; ^4^Department of Psychiatry, Depression Center, Samsung Medical Center, Sungkyunkwan University School of Medicine, Seoul, South Korea; ^5^Department of Social and Preventive Medicine, Sungkyunkwan University School of Medicine, Suwon, South Korea; ^6^Department of Health Sciences & Technology, Department of Medical Device Management & Research, and Department of Clinical Research Design & Evaluation, Samsung Advanced Institute for Health Sciences & Technology (SAIHST), Sungkyunkwan University, Seoul, South Korea

**Keywords:** method of suicide, suicide method, risk factor, suicide, suicide victims

## Abstract

**Background:** Because the suicide mortality depends on the lethality of suicide methods, the identification and prediction of suicide methods are important for suicide prevention.

**Methods:** Examination data of suicide decedents were collected based on police reports. Suicide decedents were divided into groups according to the suicide methods (hanging, gas poisoning, pesticide poisoning, jumping, drug poisoning, and drowning) they used. Predictive factors for each suicide method in comparison to other suicide methods were identified.

**Results:** Among 23,647 subjects, hanging was the most common method of suicide. Regarding gas poisoning, the history of previous suicide attempt was a risk factor and being age of 65 or older was a protective factor. Being age of 65 or older showed a highly strong association with suicide by pesticide poisoning. Being age of 18 or younger and the presence of schizophrenia were associated with jumping. A history of psychiatric outpatient treatment was a risk factor for drug poisoning. Regarding suicide by drowning, schizophrenia was a risk factor, while being age of 65 or older was a protective factor.

**Limitations:** Only eight out of a total of 17 regions in South Korea were examined and included in the data of this study. Also, the methods of suicide were defined as one method that directly caused the death, which could undermine other less fatal methods used.

**Conclusions:** There were differences in predictive factors according to the method of suicide. Predicting the method of suicide in people at high risk for suicide stands to be an important strategy for suicide prevention in clinical settings.

## Introduction

Suicide is a major cause of death worldwide, with ~800,000 people dying by suicide every year. In 2018, the incidence of suicide in South Korea was 26.6 per 100,000 people (1), which is the highest national incidence among the Organization for Economic Cooperation and Development (OECD) countries (2). Although the government of South Korea has implemented suicide prevention policies, including the enactment of the Suicide Prevention Law, the positive effects of government actions are still insufficient.

The lethality of suicide methods affects suicide mortality. Therefore, the identification and prediction of suicide methods are important for suicide prevention. The process and mechanism of choosing a suicide method are complex and multidetermined, influenced by various factors, such as environment, culture, as well as individual characteristics. According to previous studies, the differences regarding methods of suicide appear between countries and regions (3–7). Trends in suicide methods may change with time (8, 9) as new methods of suicide emerge (10) and the diffusion occur between one population and another population (11). The selection of suicide method is also known to be affected by season or day of the week (7), media reports (12), genetic effect (13), and comorbid physical or psychiatric disorders (14, 15). High accessibility, such as specific drug use, household firearm ownership, occupational drug use, also acts as predictive factors for suicide methods (15–17). Moreover, previous studies have reported that sex, education, marital status, residential areas, leaving a suicide note, and experiencing interpersonal conflict are associated with the method of suicide (12, 18–20).

Nevertheless, the evidence on risk factors according to suicide methods has been established in small sample sizes in few countries, with known limitations that comparative analysis of suicide methods is insufficient. In this study, we aim to investigate the comprehensive risk factors of suicide methods using the examination data of police reports on people who have died by suicide in South Korea.

## Methods

### Data Source and Study Population

This study used data from the Korean National Investigations of Suicide Victims (the KNIGHTS study) conducted by the Korea Psychological Autopsy Center (KPAC) (21). The KNIGHTS study was conducted by examining police investigation reports of people who died by suicide from January 1, 2013 to December 31, 2017. Trained investigators comprising mental health professionals (including certified psychiatric and mental health nurses), mental health psychologists, and mental health social workers with experience in psychiatric epidemiologic surveys were recruited for the study. The team visited a total of 254 police stations in 17 regions of South Korea. They examined police reports on people who had died by suicide and identified basic personal information, information related to the suicide, information on the causes of suicide, and information from informants' interview. According to the Korea National Statistical Office, the number of people who died by suicide during the study period was estimated to be ~70,000. As of March 2020, KPAC had completed the examinations in eight regions (Seoul, Sejong, Daejeon, Gwangju, Jeju, Gangwon-do, Chungcheongbuk-do, and Chungcheongnam–do) of the 17 regions. At that time, data were publicly available on 23,648 deaths by suicide. The data of the KNIGHTS study were categorized into general disclosure, limited disclosure, and non-disclosure according to their characteristics, and we were able to include variables with general disclosure and limited disclosure in the analyses. The analyses of this data form the basis of the current study. Informed consent was waived by the Institutional Review Board of Samsung Medical Center because the study population is deceased.

### Methods of Suicide

To identify the characteristics of suicide decedents according to the suicide method, we investigated the methods of suicide in the study population. Per KNIGHTS study protocol, the method of suicide was recorded as the one method that resulted in death. When two or more methods were used for suicide, the method of suicide was recorded as the direct cause of death based on police reports. When two or more direct causes of death were reported by police, the method with the higher fatality was recorded as the method of suicide.

### Outcomes

We identified demographic, suicide-related, and psychiatric characteristics of people who died by suicide. The demographic characteristics included sex, age, employment status, and the presence of physical illness. The suicide-related characteristics included the location of suicide, drinking status, joint suicide, homicide-suicide, the presence of a suicide note, and major cause of suicide. The psychiatric characteristics included psychiatric symptoms, psychiatric diagnosis, psychiatric treatment, the history of previous suicide attempts, and the history of previous non-suicidal self-injuries. Regarding the potential risk factors, we investigated the association between potential risk factors and each method of suicide in comparison to other methods of suicide.

### Statistical Analyses

We investigated the distribution of demographic, suicide-related, and psychiatric characteristics, of suicide decedents according to various methods of suicide. Comparisons between methods of suicide were conducted using chi-squared tests. The multivariate logistic regression analyses were used to calculate the odds ratios (ORs) for potential risk factors associated with each method of suicide in comparison to other reported methods of suicide. All statistical analyses were performed using SAS software version 9.4 (SAS Institute Inc., Cary, NC, USA).

## Results

### Methods of Suicide in All Subjects

Among 23,652 people who died by suicide, 12,283 (51.93%) died by hanging, which was the most common method, followed by jumping (15.81%), gas poisoning (13.73%), pesticide poisoning (8.00%), drowning (5.25%), and drug poisoning (2.10%). We subsequently analyzed the six methods of suicide including hanging, gas poisoning, pesticide poisoning, jumping, drug poisoning, and drowning. Four of the suicide cases by hanging were excluded due to insufficient personal information about the victims.

### Demographic and Suicide Characteristics in Subjects

Table 1 shows the demographic characteristics and suicide-related information of the subjects. Males accounted for 69.7% of the sample. Although more males comprised the total sample, in cases of suicide due to drug poisoning, females accounted for 48.3% of deaths and the sex differences were small. The age distribution of the subjects also showed differences according to the method of suicide. Nearly half of the subjects aged 10–19 years died by jumping (49.8%), and the subjects aged 70 or older accounted for 54.9% of suicide deaths by pesticide poisoning. More specifically, 65.9% of subjects who died by pesticide poisoning and 51.7% of subjects who died by drug poisoning had physical illnesses.

**Table 1:** Demographic and suicide characteristics of people who died by suicide.

	**Total people who died by suicide (*****n*** **=** **23,648)**		**Method of suicide**												
			**Hanging (*****n*** **=** **12,279)**		**Gas poisoning (*****n*** **=** **3,248)**		**Pesticide poisoning (*****n*** **=** **1,893)**		**Jumping (*****n*** **=** **3,739)**		**Drug poisoning (*****n*** **=** **497)**		**Drowning (*****n*** **=** **1,241)**		***p***
	***N***	**%**	***N***	**%**	***N***	**%**	***N***	**%**	***N***	**%**	***N***	**%**	***N***	**%**	
Sex															<0.0001
Male	16,485	69.71	8,762	71.36	2,602	80.11	1,298	68.57	2,125	56.83	257	51.71	932	75.1	
Female	7,163	30.29	3,517	28.64	646	19.89	595	31.43	1,614	43.17	240	48.29	309	24.9	
Age (years)															<0.0001
10–19	508	2.15	132	1.08	41	1.26	1	0.05	253	6.77	3	0.6	73	5.88	
20–29	2,212	9.35	910	7.41	408	12.56	8	0.42	534	14.28	38	7.65	265	21.35	
30–39	3,682	15.57	1,856	15.12	874	26.91	44	2.32	546	14.6	76	15.29	212	17.08	
40–49	4,443	18.79	2,363	19.24	926	28.51	133	7.03	548	14.66	113	22.74	218	17.57	
50–59	4,660	19.71	2,663	21.69	622	19.15	310	16.38	585	15.65	104	20.93	203	16.36	
60–69	3,011	12.73	1,673	13.62	238	7.33	358	18.91	430	11.5	72	14.49	116	9.35	
70–79	3,207	13.56	1,702	13.86	102	3.14	612	32.33	507	13.56	64	12.88	94	7.57	
≥ 80	1,925	8.14	980	7.98	37	1.14	427	22.56	336	8.99	27	5.43	60	4.83	
Employment status															<0.0001
Employed	5,007	21.17	2,755	22.44	998	30.73	159	8.4	585	15.65	78	15.69	271	21.84	
Self-employed	2,985	12.62	1,721	14.02	517	15.92	366	19.33	207	5.54	37	7.44	71	5.72	
Student	806	3.41	241	1.96	99	3.05	1	0.05	329	8.8	8	1.61	114	9.19	
Housewife	1,146	4.85	649	5.29	55	1.69	54	2.85	279	7.46	28	5.63	39	3.14	
Unemployed	11,943	50.5	6,026	49.08	1,251	38.52	1,199	63.34	2,139	57.21	292	58.75	610	49.15	
Other	1,761	7.45	887	7.22	328	10.1	114	6.02	200	5.35	54	10.87	136	10.96	
Physical illness															<0.0001
Yes	9,427	39.86	5,024	40.92	753	23.18	1,247	65.87	1,442	38.57	257	51.71	354	28.53	
No	7,721	32.65	4,007	32.63	1,345	41.41	309	16.32	1,254	33.54	122	24.55	491	39.56	
Unknown	6,500	27.49	3,248	26.45	1,150	35.41	337	17.8	1,043	27.9	118	23.74	396	31.91	
Physical disability															<0.0001
Yes	1,304	5.51	657	5.35	118	3.63	154	8.14	236	6.31	31	6.24	48	3.87	
No	11,702	49.48	6,170	50.25	1,675	51.57	806	42.58	1,840	49.21	235	47.28	632	50.93	
Unknown	10,642	45	5,452	44.4	1,455	44.8	933	49.29	1,663	44.48	231	46.48	561	45.21	
Location of suicide															<0.0001
Home	13,087	55.34	7,875	64.13	1,604	49.38	1,469	77.60	1,283	34.31	361	72.64	8	0.64	
Home of	334	1.41	154	1.25	64	1.97	22	1.16	72	1.93	10	2.01	0	0	
acquaintance															
School/work place	804	3.40	608	4.95	74	2.28	50	2.64	42	1.12	7	1.41	2	0.16	
Public place	6,416	27.13	2,106	17.15	1,135	34.94	216	11.41	2,125	56.83	47	9.46	652	52.54	
Accommodations	1,112	4.70	650	5.29	272	8.37	42	2.22	45	1.20	53	10.66	0	0	
Suburbs/hill	865	3.66	691	5.63	65	2	59	3.12	14	0.37	11	2.21	3	0.24	
Hospital	301	1.27	117	0.95	1	0.03	10	0.53	155	4.15	2	0.4	0	0	
Other	729	3.08	78	0.64	33	1.02	25	1.32	3	0.08	6	1.21	576	46.41	
Drinking status															<0.0001
Drinker	6,619	27.99	3,234	26.34	1,435	44.18	570	30.11	669	17.89	215	43.26	292	23.53	
Non-drinker	7,666	32.42	3,943	32.11	673	20.72	644	34.02	1,670	44.66	148	29.78	281	22.64	
Unknown	9,363	39.59	5,102	41.55	1,140	35.1	679	35.87	1,400	37.44	134	26.96	668	53.83	
Joint suicide															<0.0001
Yes	327	1.38	28	0.23	251	7.73	18	0.95	13	0.35	1	0.2	10	0.81	
No	23,220	98.19	12,237	99.66	2,996	92.24	1,873	98.94	3,726	99.65	495	99.6	1,148	92.51	
Unknown	101	0.43	14	0.11	1	0.03	2	0.11	0	0	1	0.2	83	6.69	
Homicide-suicide															<0.0001
Yes	92	0.39	41	0.33	4	0.12	11	0.58	13	0.35	1	0.2	4	0.32	
No	23,286	98.47	12,116	98.67	3,197	98.43	1,861	98.31	3,710	99.22	491	98.79	1,183	95.33	
Unknown	270	1.14	122	0.99	47	1.45	21	1.11	16	0.43	5	1.01	54	4.35	
Suicide note															<0.0001
Yes	8,576	36.27	4,626	37.67	1,804	55.54	399	21.08	923	24.69	235	47.28	340	27.4	
No	12,706	53.73	6,486	52.82	1,236	38.05	1,239	65.45	2,372	63.44	218	43.86	748	60.27	
Unknown	2,366	10.01	1,167	9.5	208	6.4	255	13.47	444	11.87	44	8.85	153	12.33	
Major cause of suicide															<0.0001
Occupational	1,080	4.57	600	4.89	149	4.59	28	1.48	179	4.79	5	1.01	93	7.49	
Economic	4,410	18.65	2,419	19.7	1,207	37.16	152	8.03	271	7.25	51	10.26	224	18.05	
Family-related	2,500	10.57	1,443	11.75	307	9.45	259	13.68	276	7.38	48	9.66	99	7.98	
Interpersonal	1,154	4.88	639	5.2	205	6.31	45	2.38	148	3.96	16	3.22	65	5.24	
Physical health	4,235	17.91	2,298	18.71	233	7.17	677	35.76	672	17.97	76	15.29	120	9.67	
Mental health	9,018	38.13	4,265	34.73	938	28.88	661	34.92	2,037	54.48	282	56.74	511	41.18	
Other	397	1.68	193	1.57	76	2.34	21	1.11	58	1.55	2	0.4	31	2.5	
Unknown	854	3.61	422	3.44	133	4.09	50	2.64	98	2.62	17	3.42	98	7.9	

Suicide characteristics also differed according to the method of suicide. More than half of the subjects who died in hospitals (51.5%) had selected jumping as the method. The status of drinking alcohol at the time of death was a common factor in a significant percentage of subjects who died by gas poisoning (44.2%) and in subjects who died by drug poisoning (43.3%). In the case of suicide by gas poisoning, the percentage of those who consumed alcohol (44.2%) was more than twice the percentage of those who did not drink at the time of death (20.7%). Of people who elected joint suicide, 76.8% died by gas poisoning. Of people who died by gas poisoning, 55.5% wrote suicide notes before death. In 54.5 and 56.7% of people who died by jumping and by drug poisoning, respectively, the major cause of suicide was mental health problems.

### Psychiatric Characteristics of People Who Died by Suicide

**Figure 1:**
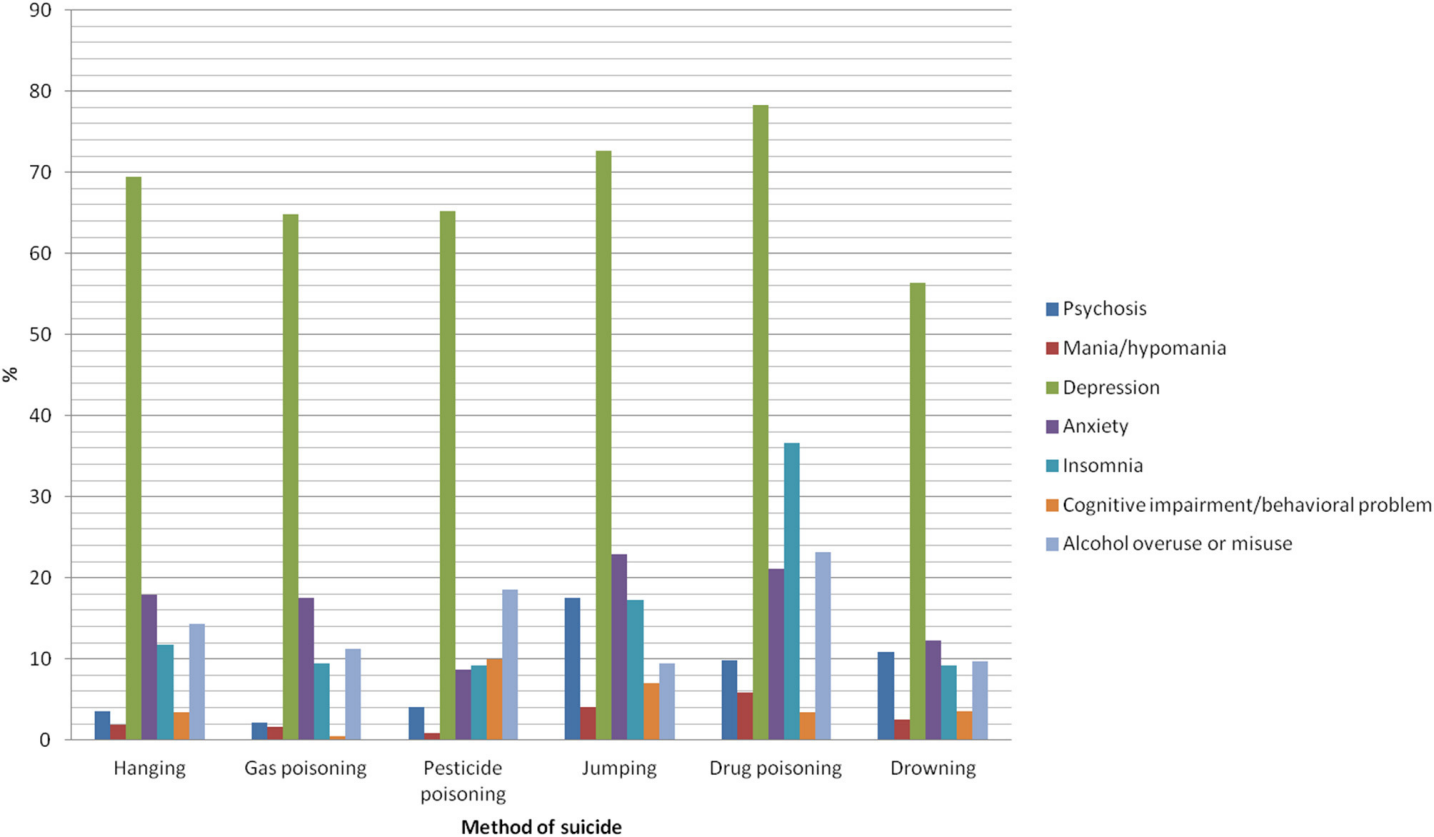
Manifestation of psychiatric symptoms before death by suicide according to suicide method.

Table 2 shows the psychiatric characteristics of people who died by suicide. Among 23,648 subjects, 84.0% had psychiatric symptoms and 68.3% had symptoms of depression. Psychiatric symptoms were present in 94.8 and 90.3% of people who died by drug poisoning and by jumping, respectively. Of subjects with psychosis, 43.9% died by jumping. Among the subjects who died by drug poisoning, 36.6% had insomnia, with this psychiatric symptom having the highest proportion in this group compared to those who died by other methods of suicide (Figure 1). Having a known history of psychiatric diagnosis was more frequent among subjects in the jumping group (50.1%) and in the drug poisoning group (64.0%), with 50.4 and 62.8% of the subjects having a history of psychiatric treatment, respectively. In these two groups, the most common psychiatric diagnosis was depressive disorders. 28.48 and 37.83% of subjects in the jumping group and the drug poisoning group, respectively, had been diagnosed with depressive disorders. Among the people diagnosed with schizophrenia, 49.51% died by jumping. Among the subjects in the drug poisoning group, there were more people with a history of attempted suicide (27.6%) than those without (18.9%).

**Table 2:** Psychiatric characteristics of people who died by suicide.

	**Total people who died by suicide (*****n*** **=** **23,648)**		**Method of suicide**												
			**Hanging (*****n*** **=** **12,279)**		**Gas poisoning (*****n*** **=** **3,248)**		**Pesticide poisoning (*****n*** **=** **1,893)**		**Jumping (*****n*** **=** **3,739)**		**Drug poisoning (*****n*** **=** **497)**		**Drowning (*****n*** **=** **1,241)**		***p***
	***N***	**%**	***N***	**%**	***N***	**%**	***N***	**%**	***N***	**%**	***N***	**%**	***N***	**%**	
Presence of psychiatric symptoms															<0.0001
Yes	19,872	84.03	10,261	83.57	2,607	80.26	1,574	83.15	3,377	90.32	471	94.77	956	77.03	
No	674	2.85	378	3.08	66	2.03	45	2.38	91	2.43	3	0.6	72	5.8	
Unknown	3,102	13.12	1,640	13.36	575	17.7	274	14.47	271	7.25	23	4.63	213	17.16	
**Psychiatric symptoms**															
Psychosis	1,495	6.32	436	3.55	69	2.12	76	4.01	656	17.54	49	9.86	135	10.88	<0.0001
Mania/hypomania	531	2.25	229	1.86	55	1.69	17	0.90	151	4.04	29	5.84	32	2.58	<0.0001
Depression	16,154	68.31	8,534	69.50	2,105	64.81	1,235	65.24	2,716	72.64	389	78.27	700	56.41	<0.0001
Anxiety	4,173	17.65	2,196	17.88	569	17.52	165	8.72	857	22.92	105	21.13	153	12.33	<0.0001
Insomnia	2,985	12.62	1,439	11.72	309	9.51	175	9.24	648	17.33	182	36.62	114	9.19	<0.0001
Cognitive impairment/behavioral problem	978	4.14	416	3.39	17	0.52	189	9.98	264	7.06	17	3.42	44	3.55	<0.0001
Alcohol overuse or misuse	3,185	13.47	1,762	14.35	366	11.27	352	18.59	351	9.39	115	23.14	121	9.75	<0.0001
Overuse or misuse of other substances	299	1.26	162	1.32	43	1.32	17	0.90	40	1.07	15	3.02	18	1.45	0.0125
Behavioral addiction	763	3.23	394	3.21	204	6.28	19	1.00	66	1.77	12	2.41	49	3.95	<0.0001
Pediatric psychiatric symptoms	82	0.35	21	0.17	5	0.15	3	0.16	40	1.07	1	0.2	10	0.81	<0.0001
History of psychiatric diagnosis															<0.0001
Yes	7,392	31.26	3,321	27.05	651	20.04	519	27.42	1,874	50.12	318	63.98	434	34.97	
No	6,185	26.15	3,463	28.20	883	27.19	475	25.09	795	21.26	55	11.07	341	27.48	
Unknown	10,071	42.59	5,495	44.75	1,714	52.77	899	47.49	1,070	28.62	124	24.95	466	37.55	
**Psychiatric diagnosis**															
Schizophrenia	719	3.04	163	1.33	21	0.65	34	1.80	356	9.52	26	5.23	84	6.77	<0.0001
Bipolar disorder	401	1.70	160	1.3	30	0.92	15	0.79	133	3.56	24	4.83	28	2.26	0.0004
Depressive disorders	4,538	19.19	2,182	17.77	413	12.72	297	15.69	1,065	28.48	188	37.83	235	18.94	<0.0001
Anxiety disorders	660	2.79	305	2.48	60	1.85	27	1.43	177	4.73	35	7.04	29	2.34	0.0127
Adjustment disorder	60	0.25	28	0.23	6	0.18	0	0.00	15	0.4	2	0.4	5	0.4	0.2208
Somatic symptom disorder	34	0.14	15	0.12	3	0.09	3	0.16	10	0.27	2	0.4	1	0.08	0.9366
Sleep disorders	848	3.59	400	3.26	91	2.8	60	3.17	177	4.73	54	10.87	33	2.66	<0.0001
Neurocognitive disorders	518	2.19	210	1.71	5	0.15	105	5.55	143	3.82	8	1.61	29	2.34	<0.0001
Alcohol use disorders	733	3.10	351	2.86	64	1.97	66	3.49	137	3.66	43	8.65	34	2.74	<0.0001
Other substance-related disorders	12	0.05	8	0.07	0	0.00	0	0.00	2	0.05	0	0	0	0	0.7444
Behavioral addiction	14	0.06	3	0.02	6	0.18	0	0.00	4	0.11	0	0	1	0.08	0.0075
Pediatric psychiatric disorder	32	0.14	6	0.05	2	0.06	2	0.11	21	0.56	0	0	1	0.08	0.0001
Other	206	0.87	83	0.68	15	0.46	9	0.48	76	2.03	6	1.21	12	0.97	0.014
History of psychiatric treatment															<0.0001
Yes	7,333	31.01	3,279	26.7	644	19.83	496	26.2	1,886	50.44	312	62.78	440	35.46	
No	6,278	26.55	3,518	28.65	896	27.59	481	25.41	811	21.69	55	11.07	337	27.16	
Unknown	10,037	42.44	5,482	44.65	1,708	52.59	916	48.39	1,042	27.87	130	26.16	464	37.39	
**Psychiatric treatment**															
Outpatient clinic	5,931	25.08	2,617	21.31	493	15.18	377	19.92	1,613	43.14	257	51.71	349	28.12	0.3185
Admission	2,046	8.65	745	6.07	124	3.82	109	5.76	725	19.39	80	16.1	155	12.49	<0.0001
Treatment in other departments	295	1.25	140	1.14	15	0.46	22	1.16	82	2.19	19	3.82	7	0.56	0.0101
Counseling center	142	0.60	64	0.52	17	0.52	2	0.11	35	0.94	4	0.8	18	1.45	0.0016
Other	60	0.25	29	0.24	5	0.15	3	0.16	17	0.45	2	0.4	3	0.24	0.9991
Previous suicide attempt															<0.0001
Yes	3,986	16.86	1,952	15.90	610	18.78	211	11.15	713	19.07	137	27.57	207	16.68	
No	6,221	26.31	3,224	26.26	766	23.58	545	28.79	1,075	28.75	94	18.91	338	27.24	
Unknown	13,441	56.84	7,103	57.85	1,872	57.64	1,137	60.06	1,951	52.18	266	53.52	696	56.08	
Previous self-injury															<0.0001
Yes	903	3.82	451	3.67	99	3.05	17	0.9	192	5.14	30	6.04	55	4.43	
No	7,039	29.77	3,607	29.38	937	28.85	586	30.96	1,203	32.17	125	25.15	369	29.73	
Unknown	15,706	66.42	8,221	66.95	2,212	68.1	1,290	68.15	2,344	62.69	342	68.81	817	65.83	

### Adjusted Odds Ratios of Potential Risk Factors for Each Method of Suicide

Table 3 shows adjusted odds ratios of potential risk factors for each method of suicide in comparison to other methods of suicide. Regarding hanging, in comparison to subjects aged 19–34 years, being 18 years of age or younger (OR 0.49, 95% CI: 0.33–0.71) and having schizophrenia (OR 0.38, 95% CI: 0.30–0.47) were protective factors. Being 35–49 years of age (OR 1.43, 95% CI: 1.27–1.61), 50–64 years of age (OR 1.60, 95% CI: 1.41–1.81), and 65 years of age or older (OR 1.50, 95% CI: 1.31–1.72) were risk factors for suicide by hanging in comparison to being 19–34 years of age. Regarding gas poisoning, being 65 years of age or older (OR 0.19, 95% CI: 0.15–0.24) was a protective factor, while having previously attempted suicide was a risk factor (OR 1.62, 95% CI: 1.33–1.98). Compared to being 19–34 years of age, being 35 years of age or older showed a strong association with pesticide poisoning. Odds ratios for subjects in age groups of 35–49, 50–64, and 65 years or older were 2.81 (95% CI: 1.55–5.11), 10.43 (95% CI: 5.92–18.38), and 22.97 (95% CI: 12.97–40.67), respectively. Being self-employed (OR 2.16, 95% CI: 1.76–2.66), having schizophrenia (OR 2.31, 95% CI: 1.49–3.57), and having alcohol use disorders (OR 2.63, 95% CI: 1.86–3.71) were also risk factors for suicide by pesticide poisoning. Being a housewife was a protective factor for suicide by pesticide poisoning (OR 0.48, 95% CI: 0.32–0.72). Regarding suicide by jumping, being 18 years of age or younger (OR 3.71 95% CI: 2.63–5.22) and having schizophrenia (OR 2.39, 95% CI: 1.96–2.93) were risk factors, while being self-employed was a protective factor (OR 0.46, 95% CI: 0.37–0.57). Regarding suicide by drug poisoning, alcohol use disorders (OR 2.19, 95% CI: 1.49–3.24) and psychiatric treatment in outpatient clinics (OR 2.86, 95% CI: 1.92–4.26) were risk factors, while being 65 years of age or older (OR 0.49, 95% CI: 0.31–0.77) was a protective factor. Regarding suicide by drowning, having schizophrenia (OR 1.69, 95% CI: 1.21–2.36) was a risk factor and being 65 years of age or older (OR 0.32, 95% CI: 0.24–0.42) was a protective factor.

**Table 3:** Adjusted odds ratios of potential risk factors for each method of suicide.

	**Hanging (*n* = 12,279)**	**Gas poisoning (*n* = 3,248)**	**Pesticide poisoning (*n* = 1,893)**	**Jumping *n* = 3,739)**	**Drug poisoning (*n* = 497)**	**Drowning (*n* = 1,241)**
	**OR (95% CI)**	**OR (95% CI)**	**OR (95% CI)**	**OR (95% CI)**	**OR (95% CI)**	**OR (95% CI)**
Female sex	0.91 (0.84–0.99)^*^	0.63 (0.54–0.73)^***^	1.50 (1.28–1.77)^***^	1.40 (1.26–1.56)^***^	1.74 (1.35–2.25)^***^	0.59 (0.49–0.72)^***^
**Age (years)**						
≤ 18	0.49 (0.33–0.71)^***^	0.32 (0.16–0.64)^**^	0.00 (<0.01–>999.99)	3.71 (2.63–5.22)^***^	0.00 (<0.01–>999.99)	0.94 (0.58–1.52)
19–34	Reference	Reference	Reference	Reference	Reference	Reference
35–49	1.43 (1.27–1.61)^***^	1.04 (0.89–1.21)	2.81 (1.55–5.11)^***^	0.62 (0.54–0.72)^***^	1.18 (0.84–1.68)	0.50 (0.40–0.63)^***^
50–64	1.60 (1.41–1.81)^***^	0.53 (0.44–0.63)^***^	10.43 (5.92–18.38)^***^	0.63 (0.53–0.73)^***^	0.91 (0.63–1.33)	0.42 (0.33–0.54)^***^
≥65	1.50 (1.31–1.72)^***^	0.19 (0.15–0.24)^***^	22.97 (12.97–40.67)^***^	0.75 (0.63–0.89)^**^	0.49 (0.31–0.77)^**^	0.32 (0.24–0.42)^***^
**Employment status**						
Employed	1.18 (1.06–1.31)^**^	1.03 (0.89–1.20)	0.89 (0.67–1.19)	0.82 (0.70–0.94)^**^	0.86 (0.61–1.22)	0.75 (0.60–0.93)^*^
Self-employed	1.26 (1.11–1.43)^***^	1.11 (0.92–1.33)	2.16 (1.76–2.66)^***^	0.46 (0.37–0.57)^***^	0.59 (0.35–0.97)^*^	0.38 (0.26–0.55)^***^
Student	0.71 (0.55–0.92)^**^	0.62 (0.43–0.90)^*^	0.49 (0.06–3.71)	1.47 (1.13–1.90)^**^	0.61 (0.24–1.56)	1.42 (1.01–2.01)^*^
Housewife	1.36 (1.15–1.60)^***^	0.51 (0.36–0.73)^***^	0.48 (0.32–0.72)^***^	1.14 (0.94–1.38)	0.51 (0.31–0.85)^**^	0.80 (0.52–1.23)
Unemployed	Reference	Reference	Reference	Reference	Reference	Reference
Other	0.91 (0.77–1.07)	1.34 (1.04–1.69)^*^	1.19 (0.84–1.70)	0.71 (0.56–0.89)^**^	1.79 (1.21–2.64)^**^	1.20 (0.87–1.64)
Physical illness	0.99 (0.90–1.09)	0.74 (0.63–0.87)^***^	1.28 (1.05–1.56)^*^	1.02 (0.89–1.16)	1.58 (1.16–2.16)^**^	0.92 (0.73–1.14)
**Psychiatric diagnosis**						
Schizophrenia	0.38 (0.30–0.47)^***^	0.25 (0.15–0.41)^***^	2.31 (1.49–3.57)^***^	2.39 (1.96–2.93)^***^	1.02 (0.63–1.65)	1.69 (1.21–2.36)^**^
Bipolar disorder	0.94 (0.75–1.18)	0.81 (0.53–1.22)	1.06 (0.61–1.86)	1.12 (0.88–1.42)	1.50 (0.94–2.38)	0.96 (0.63–1.47)
Depressive disorders	1.04 (0.91–1.19)	1.06 (0.82–1.36)	1.00 (0.78–1.29)	0.97 (0.84–1.13)	0.99 (0.71–1.37)	1.01 (0.78–1.32)
Anxiety disorders	0.98 (0.83–1.17)	0.85 (0.62–1.16)	0.74 (0.48–1.14)	1.17 (0.96–1.42)	1.14 (0.77–1.69)	0.76 (0.51–1.14)
Sleep disorders	0.87 (0.74–1.02)	1.31 (1.00–1.72)	0.99 (0.72–1.36)	0.98 (0.81–1.19)	1.59 (1.13–2.26)^**^	0.86 (0.58–1.28)
Neurocognitive disorders	0.71 (0.57–0.88)^**^	0.23 (0.07–0.72)^*^	1.67 (1.24–2.24)^**^	1.23 (0.96–1.58)	0.70 (0.33–1.50)	1.46 (0.90–2.39)
Alcohol use disorders	1.03 (0.86–1.24)	0.91 (0.66–1.26)	2.63 (1.86–3.71)^***^	0.73 (0.59–0.92)^**^	2.19 (1.49–3.24)^***^	0.71 (0.47–1.07)
**Psychiatric treatment**						
Outpatient clinic	0.77 (0.67–0.89)^***^	0.75 (0.58–0.97)^*^	0.76 (0.58–1.00)	1.79 (1.52–2.11)^***^	2.86 (1.92–4.26)^***^	0.99 (0.75–1.31)
Admission	0.70 (0.61–0.80)^***^	0.62 (0.48–0.80)^***^	0.63 (0.47–0.85)^**^	1.92 (1.66–2.22)^***^	0.83 (0.59–1.15)	1.20 (0.93–1.56)
Other	0.98 (0.79–1.22)	0.79 (0.52–1.21)	0.56 (0.34–0.90)^*^	1.32 (1.02–1.70)^*^	1.63 (0.97–2.73)	1.12 (0.71–1.74)
Previous suicide attempt	0.97 (0.85–1.11)	1.62 (1.33–1.98)^***^	0.71 (0.54–0.94)^*^	0.75 (0.63–0.89)^**^	1.43 (0.96–2.14)	0.93 (0.70–1.24)
Previous self–injury	1.24 (1.01–1.52)^*^	0.55 (0.39–0.77)^**^	0.65 (0.36–1.20)	0.96 (0.75–1.24)	0.90 (0.52–1.58)	0.92 (0.61–1.41)

* *p < 0.05*,

** *p < 0.01*,

*** *p < 0.001*.

Table 4 presents the risk factors of each method of suicide in a descending order of ORs and protective factors of each method in an ascending order of ORs. Being 35 years of age or older, having alcohol use disorders, having schizophrenia, and being self-employed were strong risk factors for suicide by pesticide poisoning. Being 18 years of age or younger and having schizophrenia were strong risk factors for suicide by jumping. Treatment in psychiatric outpatient clinics and alcohol use disorders were strong risk factors for suicide by drug poisoning.

**Table 4:** Risk factors and protective factors for each method of suicide based on multivariate logistic regression^a^.

	**Hanging**		**Gas poisoning**		**Pesticide poisoning**		**Jumping**		**Drug poisoning**		**Drowning**	
	**Factor**	**OR**	**Factor**	**OR**	**Factor**	**OR**	**Factor**	**OR**	**Factor**	**OR**	**Factor**	**OR**
Risk factors	Age 50–64 years	1.60	Previous suicide attempt	1.62	Age ≥ 65 years	22.97	Age ≤ 18 years	3.71	Psychiatric outpatient clinic	2.86	Schizophrenia	1.69
	Age ≥ 65 years	1.50			Age 50–64 years	10.43	Schizophrenia	2.39	Alcohol use disorders	2.19	Student	1.42
	Age 35–49 years	1.43			Age 35–49 years	2.81	Admission to psychiatry	1.92	Female sex	1.74		
	Housewife	1.36			Alcohol use disorders	2.63	Psychiatric outpatient clinic	1.79	Sleep disorders	1.59		
	Self-employed	1.26			Schizophrenia	2.31	Student	1.47	Physical illness	1.58		
	Previous self-injury	1.24			Self-employed	2.16	Female sex	1.40				
	Employed	1.18			Neurocognitive disorders	1.67						
					Female sex	1.50						
Protective factors	Schizophrenia	0.38	Age ≥ 65 years	0.19	Housewife	0.48	Self-employed	0.46	Age ≥ 65 years	0.49	Age ≥ 65 years	0.32
	Age ≤ 18 years	0.49	Neurocognitive disorders	0.23	Admission to psychiatry	0.63	Age 35–49 years	0.62	Housewife	0.51	Self-employed	0.38
	Admission to psychiatry	0.70	Schizophrenia	0.25	Previous suicide attempt	0.71	Age 50–64 years	0.63	Self-employed	0.59	Age 50–64 years	0.42
	Student	0.71	Age ≤ 18 years	0.32			Alcohol use disorders	0.73			Age 35–49 years	0.50
	Neurocognitive disorders	0.71	Housewife	0.51			Age ≥ 65 years	0.75			Female sex	0.59
	Psychiatric outpatient clinic	0.77	Age 50–64 years	0.53			Previous suicide attempt	0.75			Employed	0.75
	Female sex	0.91	Student	0.62			Employed	0.82				
			Admission to psychiatry	0.62								
			Female sex	0.63								
			Physical illness	0.74								
			Psychiatric outpatient clinic	0.75								
			Previous self-injury	0.55								

a *Factors have significance at p > 0.05; detailed statistical measures of each factor are listed in Table 3*.

## Discussion

This study demonstrated that more than half of people who died by suicide chose hanging as the method of suicide. Strong risk factors for pesticide poisoning were being 35 years of age or older, having alcohol use disorders, having schizophrenia, and being self-employed. In comparison to people who died by other methods of suicide, people who died by jumping were more likely to be 18 years of age or younger and to have been diagnosed with schizophrenia. People who died by drug poisoning were more likely to have a history of treatment in psychiatric outpatient clinics and have alcohol use disorders.

A previous study based on the Korean national data showed that 5.7% of people who attempted suicide used hanging as the method of suicide (22). In the current study's sample of people who died by suicide, hanging was the most common method of suicide. This difference in findings can be attributed to the high fatality of hanging. In our study, even though the associations between suicide risk factors and hanging were not very strong, a previous history of self-injury (one strong risk factor of suicide due to hanging) had clinical significance as an important risk factor, demanding assessments in clinical settings.

The second most common suicide method was jumping. Compared to other suicide methods, people who died by jumping were more likely to be younger and had been diagnosed with schizophrenia. Considering that about half of teenagers and subjects diagnosed with schizophrenia died by jumping, clinicians should be aware of the risk of jumping in these populations. Also, with the risk of suicide being high in this population, strategies to reduce accessibility to jumping may be necessary to prevent suicide.

Pesticide poisoning accounted for 8% of suicide deaths herein. The main finding is that pesticide poisoning cases were primarily concentrated in the elderly. Although the risk of pesticide poisoning increased for people as early as age 35, the risk increased abruptly in the group of people aged 65 years or older, with the risk in this group being about 23 times the risk in people aged 19–34 years. Moreover, having been diagnosed with schizophrenia and alcohol use disorders were strong risk factors for suicide by pesticide poisoning. Therefore, physicians should be aware of the risk of suicide by pesticide poisoning in people with these disorders and in the elderly and should evaluate at-risk people's access to pesticides (including uses for work).

Drug poisoning accounted for 2% of suicide deaths among the subjects herein. In a previous study of people who attempted suicide in South Korea, drug poisoning was the most common method, accounting for 53.7% of all suicide attempts (22). The low lethality of drug poisoning may explain such difference. In assessing the characteristics of the people who died by drug poisoning, females accounted for 48%, the highest proportion among all methods of suicide. Subjects who died by drug poisoning were more likely to have physical illnesses and psychiatric symptoms. Among comorbid psychiatric symptoms (while depression was the most common symptom), insomnia showed a large proportion in those who died by drug poisoning compared to other methods of suicide. Treatment in psychiatric outpatient clinics was a strong risk factor for suicide by drug poisoning. This result is consistent with the findings of previous studies. A cross-sectional study conducted in Japan showed that poisoning by prescription drugs was used more frequently for suicide in people who saw a psychiatrist than in people who did not see a psychiatrist (23). These findings, however, should be interpreted with caution. Physicians and psychiatrists should not undertreat patients with psychiatric symptoms due to fear of patients dying by suicide using prescribed drugs. According to our results, 96% of the subjects with a history of treatment in psychiatric outpatient clinics died by suicide methods other than drug poisoning. Moreover, depression and insomnia are independent risk factors for suicide (24–28). Therefore, for psychiatric patients with a high risk of suicide, active intervention for symptoms, short intervals of prescription drug use, and regular evaluations of suicide risk stand to be more helpful for the prevention of suicide rather than limiting the prescription of drugs.

As mentioned above, there are significant differences in the proportions of methods for suicide and suicide attempt. Although suicide attempts and completed suicide are different phenomena, the history of a suicide attempt is one of the strongest predictors for completed suicide (29) and about 10–15% of those who attempt suicide eventually die by suicide (30). It is not well-known whether suicide attempters use the same methods when they subsequently die by suicide. However, a cohort study showed that although suicide attempters are likely to choose the same method, they will select a more lethal method when they change the method of suicide (31). Moreover, a psychological autopsy study reported that the majority of the suicide attempters switched to a different method for their final act that led to the death and this suggests that people who have attempted suicide are likely to change from previous methods with low lethality to those with high lethality (32). These imply that a person who survives after a suicide attempt by a method of low lethality should be considered at high risk for a subsequent fatal suicide attempt, and appropriate intervention should be taken to prevent suicide death.

In the results, about 70% of suicide victims were male, which is consistent with the results of previous psychological autopsy studies (32, 33). This difference in sex may be related to the tendency of men to choose more violent and fatal methods when attempting suicide (34). According to a previous study, about 62% of men died by suicide at their first attempt compared to 38% in women (33). It is not clear why men choose the methods with high lethality, but there has been a wide range of explanations, including the stronger intent to die (35), being less avoidant for disfiguring wounds (36) and biological factors such as lower brain serotonin level (37).

The strength of this study is the identification of demographic and clinical risk factors for methods of suicide using a large sample of examinations based on police reports conducted according to a validated and systematic protocol. Nevertheless, this study has several limitations. First, because this study is conducted using the populations of eight regions (out of a total of 17 regions) in South Korea, it is difficult to apply the results directly to other regions or countries or to generalize findings. Trends in suicide methods may change due to the accessibility to materials and sociocultural environments, which vary with regions and time. However, our results on risk factors for methods of suicide are generally consistent with the findings of previous studies conducted in various regions and countries. This suggests that specific characteristics are shared in the process of selecting a method of suicide, suicidal intent, acceptability of a suicide method, and psychosocial factors pertaining to access to certain materials and/or methods of suicide. Second, in this study, “methods of suicide” were recorded as the direct cause of death when a person used two or more methods for suicide. In addition, when two or more direct causes of death existed, the method with the higher fatality was recorded as the method of suicide. For example, when a person died by hanging after overdosing on drugs, per the KNIGHTS study protocol, the method of suicide was recorded as hanging. Therefore, it is possible that when two or more methods were used for suicide, methods with lower fatality were underestimated. Third, several important confounders that could affect the methods of suicide were not included in the analyses because data source does not include them or they were categorized into non-disclosure variables. For example, although several personal factors including level of education (18) and religious belief (38) are known to affect the selection of suicide method, we were not able to use them in the analyses. In addition, although the data of the KNIGHTS study included information on whether a suicide victim had attempted suicide before the suicide death, the information on the method of previous suicide attempts was not included.

The possibility of a switch to another suicide method should be considered in strategies for suicide prevention by predicting and intervening in the method of suicide. A controlled trial conducted in Hong Kong demonstrated that limiting retail access to charcoal has efficacy in preventing suicide by charcoal burning. Moreover, the overall suicide rate decreased with limited retail access to charcoal (27). This suggests that limiting access to materials used in specific suicide methods does not lead potential victims to immediately switch to alternative methods of suicide. When access channels to materials that can be used in suicide are clear and when intervention is possible—as in the case of charcoal—it is necessary to limit access to the purchasing of materials intended for suicide. For example, the removal of all charcoal packs from open shelves in major retail outlets, following the Hong Kong study, stands to be efficacious not only for a reduction in suicides using gas poisoning but also for the overall prevention of suicides by all methods.

In conclusion, there are differences in demographic and clinical risk factors according to methods of suicide. Strong risk factors for differing methods of suicide include being elderly for pesticide poisoning, being a teenager and having schizophrenia for jumping, and having treatment history in psychiatric clinics for drug poisoning. Predicting methods of suicide in people at high risk for suicide through evaluation in the accessibility related to individual socio-environmental factors and acceptability is may be an important and efficacious strategy for suicide prevention in clinical settings.

## Data Availability Statement

Publicly available datasets were analyzed in this study. This data can be found at: https://data.psyauto.or.kr.

## Ethics Statement

The studies involving human participants were reviewed and approved by Institutional Review Board of Samsung Medical Center. Written informed consent from the participants' legal guardian/next of kin was not required to participate in this study in accordance with the national legislation and the institutional requirements.

## Author Contributions

HK contributed to the search for background literature, to writing the original draft of the manuscript, to reviewing, and to editing the subsequent manuscript revisions. GL and JC collected data. YK and M-HS contributed to formal analysis. HJ contributed to conceptualization, project administration, and supervision. All authors contributed to writing and editing the manuscript.

## Conflict of Interest

The authors declare that the research was conducted in the absence of any commercial or financial relationships that could be construed as a potential conflict of interest.
